# Sex- and Age-Dependent Neuroimmune Dysregulation and Early Neurodegenerative Signatures Following SARS-CoV-2 Infection in Golden Syrian Hamsters

**DOI:** 10.21203/rs.3.rs-9130692/v1

**Published:** 2026-04-07

**Authors:** Narendra Kumar, Arpan Acharya, Rajesh Das, Urvinder Kaur Sardarni, Olasunkanmi Israel Tayo, Swathi Priyanka Murakonda, Debapriya Sutar, Amouda Venkatesan, Dinakara Rao Ampasala, Samuel M. Cohen, Mystera M. Samuelson, Balasrinivasa R. Sajja, Ulf Dettmer, Nagendran Ramalingam, Hitendra S. Chand, Siddappa N. Byrareddy

**Affiliations:** 1 Department of Pharmacology and Experimental Neuroscience, University of Nebraska Medical Center, Omaha, NE, USA.; 2 Department of Bioinformatics, School of Life Sciences, Pondicherry University, Puducherry 605014, India.; 3 Sylvia Havlik Centennial Professor of Oncology, Department of Pathology, Microbiology, and Immunology and the Buffett Cancer Center, University of Nebraska Medical Center, Omaha, NE, USA.; 4 Department of Environmental, Agricultural, and Occupational Health, The University of Nebraska Medical Center, Omaha, Nebraska, USA; 5 Department of Radiology, University of Nebraska Medical Center, Omaha, NE, USA; 6 Ann Romney Center for Neurologic Diseases, Brigham and Women’s Hospital and Harvard Medical School, Boston, MA 02115, USA; 7 Department of Cellular and Molecular Medicine, Herbert Wertheim College of Medicine, Florida International University, Miami, Florida, USA

**Keywords:** Long-COVID, PASC, GSH, Neuroinflammation, Animal model, Sex-differences

## Abstract

Post-acute sequelae of SARS-CoV-2 infection, or Long-COVID, affects millions globally and is characterized by persistent symptoms affecting multiple organs, yet the underlying mechanisms remain poorly defined. Here, we used Golden Syrian Hamsters (GSH) infected with the SARS-CoV-2 to investigate how sex and age shape viral persistence, organ-specific pathology, immune responses, and neurological outcomes during acute infection and Long-COVID. We show that during Long-COVID, viral RNA persists only in the lungs of male hamsters. Lung pathology revealed sustained inflammation and tissue remodeling, with young females exhibiting greater fibrosis. Transcriptomic profiling across brain, lung, and heart identified pronounced sex- and age-dependent regulation of gene expression spanning immune, neuroinflammatory, and neurotransmitter signaling pathways. These transcriptomic alterations were accompanied by sex-specific behavioral changes and persistent microstructural remodeling in cognition-associated brain regions. Additionally, SARS-CoV-2 altered α-synuclein homeostasis and microglial activation alongside gut microbiome composition in a sex- and age-dependent manner. Together, our findings demonstrate that, in GSH Long-COVID is strongly modulated by sex and age, influencing viral RNA persistence, immune and neurobiological responses, and gut microbiota composition mirroring clinical outcomes reported in human cohorts. This study establishes SARS-CoV-2-infected GSH as a model for dissecting the mechanisms of Long-COVID and informing targeted prevention strategies.

## Introduction

Severe acute respiratory syndrome coronavirus 2 (SARS-CoV-2) is primarily recognized as a respiratory pathogen, causing symptoms such as cough, fever, and myalgia^1–3^. However, it is now well-established that SARS-CoV-2 infection extends beyond the respiratory tract and is associated with significant neurological manifestations^1,3,4^. During the acute phase of infection, patients frequently experience anosmia, ageusia, headaches, and, in some cases, cognitive impairment ^4–6^, underscoring the virus’s capacity to affect the central nervous system (CNS).

Disease severity following SARS-CoV-2 infection is strongly influenced by host factors, particularly age, sex, and pre-existing comorbidities^7^. Older individuals and those with conditions such as hypertension, diabetes, or cardiovascular diseases are at higher risk of severe illness, multi-organ dysfunction, and mortality^7–9^. Although most individuals recover from the acute infection, a substantial proportion develop persistent symptoms that extend beyond viral clearance, a condition collectively known as Post-Acute Sequelae of SARS-CoV-2 infection (PASC) or commonly referred to as Long-COVID^6,10–12^.

Long-COVID is characterized by a heterogeneous constellation of symptoms that can persist for weeks to months after the resolution of acute infection^13^. Importantly, Long-COVID affects individuals across the spectrum of initial disease severity, including those with mild or asymptomatic infections who were never hospitalized^14,15^. Persistent disease manifestations involve multiple organs and include gastrointestinal disorders, dyspnea, chronic fatigue, sleep disruptions, endocrine dysfunction, reduced exercise tolerance, and neuropsychiatric symptoms such as anxiety, depression, and cognitive impairment^13,16^. These observations underscore Long-COVID as a complex, systemic disorder rather than a simple extension of acute respiratory disease.

Despite extensive investigation into acute COVID-19, the long-term consequences of SARS-CoV-2 infection remain poorly understood. Epidemiological studies increasingly suggest that SARS-CoV-2 infection may elevate the risk of post-infection neurological disorders, including Alzheimer’s disease (AD), Parkinson’s disease (PD), and related dementias^17,18^. The risk appears particularly pronounced in individuals with pre-existing cardiometabolic comorbidities ^18^, raising concern that SARS-CoV-2 infection may accelerate or unmask neurodegenerative processes in high risk populations^19–21^.

The mechanisms underlying Long-COVID are likely multifactorial. Proposed contributors include persistence of residual viral components within tissues, sustained immune and inflammatory dysregulation, direct tissue injury, and disruption of systemic regulatory networks such as the gut–brain axis^11,19,22^. Increasing evidence suggests that aberrant or unresolved immune activation may establish a chronic inflammatory state affecting both central and peripheral nervous systems, thereby driving prolonged symptomatology in Long-COVID patients^23–25^.

At present, much of our understanding of Long-COVID derives from observational human studies, which, while informative, are inherently limited in their ability to establish causality or define underlying mechanisms. Robust preclinical models are therefore essential for dissecting the molecular, cellular, and systemic drivers of Long-COVID. The Golden Syrian hamster (GSH) represents a highly relevant model for SARS-CoV-2 infection, as it naturally supports viral entry and replication and recapitulates key features of COVID-19, including respiratory pathology and multi-organ involvement^26–28^.

In this study, we leveraged the GSH model to characterize Long-COVID across multiple organ, with a particular focus on neurological outcomes. By integrating histopathology, transcriptomics, neurobehavioral assessments, neuroimaging, and gut microbiome profiling, we delineate persistent molecular and cellular alterations in host responses. Importantly, our findings reveal pronounced sex- and age-dependent differences that may underlie differential susceptibility to Long-COVID and its associated neurological manifestations. Together, this work provides mechanistic insight into the systemic nature of Long-COVID and establishes a preclinical framework for understanding demographic heterogeneity and developing targeted therapeutic strategies.

## Results

### Sex- and age-dependent viral persistence and pulmonary pathology in a GSH Long-COVID model

**Figure 1:**
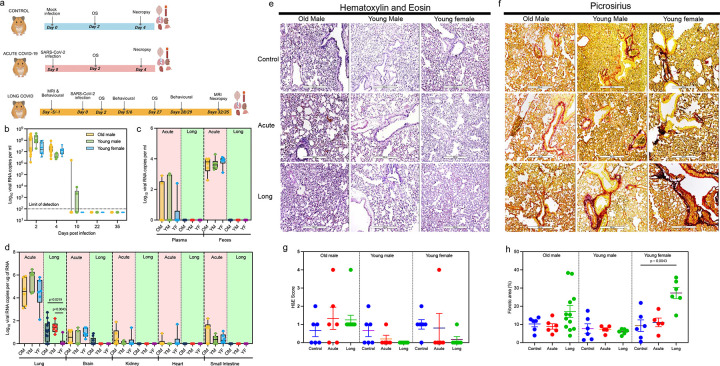
Sex- and age-dependent tissue viral persistence and pulmonary pathology after SARS-CoV-2 infection. (a) Schematic representation of experimental design. (b) Viral RNA copies in oral swabs collected at multiple time-points from all the animal groups were quantified using RT-qPCR. Dashed lines represent the limit of detection. (c-d) Viral RNA levels (Log10 copies/μg RNA or mL) quantified by droplet digital PCR in various tissues of Golden Syrian hamsters infected with SARS-CoV-2 omicron variant at acute (4 dpi) and Long (32–35 dpi) COVID timepoints. Samples were collected from the lung, brain, kidney, heart, small intestine, plasma, and feces in old male, young male, and young female hamsters. (e-f) Representative micrograph images of lung histopathology showing inflammatory and fibrotic changes post-infection, stained with (e) H&E and (f) Picrosirius red. (g) H&E histopathology scores of lung sections from control, acute, and long infection groups in OM, YM, and YF animals. (g) Fibrotic area (%) quantified using Picrosirius red staining across the same groups. Statistical comparisons between two independent groups were performed using the Mann-Whitney U test. Abbreviations: OM, old male; YM, young male; YF, young female; dpi, days post-infection.

Groups of young (8–16 wks) male and female GSHs, and the aged (36–40 wks) males were infected with the SARS-CoV-2 Omicron variant as outlined in Fig. 1a. Viral genomic RNA levels in oral swabs declined sharply and were undetectable by 10 dpi in most animals, with delayed clearance observed in two young males and one aged male until 22 dpi (Fig. 1b). Viral load was detectable in feces and plasma during acute infection (4 dpi) but fell below the limit of detection during the Long-COVID phase (30 dpi; Fig. 1c). Interestingly, during Long-COVID, SARS-CoV-2 genomic RNA persisted in the lung tissue of young and aged male hamsters, while persistence was detected only in one young female (Fig. 1d). Viral RNA levels in extrapulmonary tissues, including brain, heart, intestine, and kidney, remained low or undetectable at 30 dpi, compared with hamsters at 4 dpi (Fig. 1d).

Histological analysis revealed marked pathological changes in SARS-CoV-2-infected hamsters (Fig. 1e). Specifically, during the acute infection, lungs showed inflammatory cell infiltration and alveolar wall thickening, which persisted during the Long-COVID phase. H&E scoring demonstrated elevated inflammation in aged males at both time-points compared to controls (Fig. 1g). Severe epithelial hyperplasia was observed in one aged male each at 4 dpi and at 30 dpi, and one young female at 4 dpi (**Supplementary Fig. 1**). Picrosirius red staining showed progressive fibrotic remodeling in infected lungs over time compared to uninfected controls (Fig. 1f). Young females displayed significantly increased fibrotic area at 30 dpi (p=0.0043; Fig. 1h). Taken together, our findings indicate that pulmonary pathological outcomes following SARS-CoV-2 infection are influenced by age and sex, with young females manifesting lung fibrotic development during Long-COVID.

### Sex- and age-dependent lung transcriptional remodeling and immune responses after SARS-CoV-2 infection

**Figure 2:**
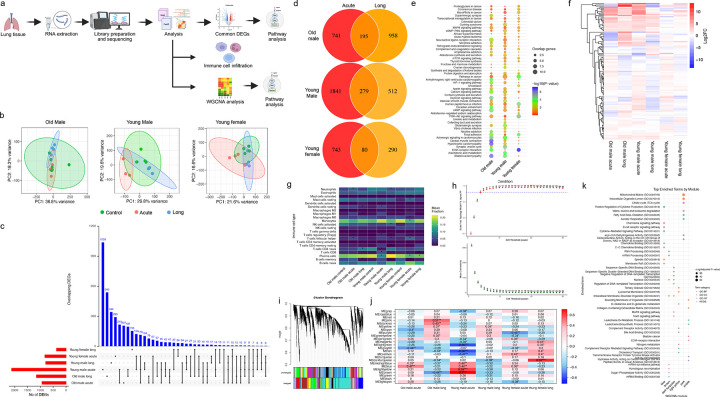
Sex- and age-dependent pathway enrichment, co-expression networks, and immune signatures in SARS-CoV-2–infected lungs. (a) Schematic representation of Lung transcriptomic analysis. (b) PCA plot showing sample clustering based on TMM-normalized gene expression. Ellipses represent group distributions for each group of animals across different conditions. The acute condition shows less variance in old males compared to the high variance observed in young males and young females. (c) Upset plot showing the overlap of differentially expressed genes (DEGs) among six experimental groups. Horizontal bars indicate total DEGs per group; vertical bars show the number of overlapping DEGs for each intersection. (d) Venn diagram showing overlap of DEGs between different experimental groups, with numbers indicating unique and shared DEGs across acute and Long-COVID. (e) Combined pathway analysis bubble plot showing significantly enriched pathways across different conditions (old male, young male, and young female). Bubble size represents the number of overlapping genes, and color intensity indicates -log10 (P-value). (f) Gene expression heatmap showing Log2 fold changes of DEGs identified across the pathways in different conditions and their status in other groups. (g) Immune cell fraction analysis showing the relative proportions of 22 different immune cell types across all experimental conditions compared to their respective controls, displayed as a heatmap with mean fraction values. (h) WGCNA soft threshold selection showing scale-free topology model fit (R^2^) and mean connectivity across different power values (1–30). The optimal soft threshold power was selected where R^2^ first exceeds the scale-free topology threshold while preserving network connectivity. (i) Gene clustering dendrogram showing WGCNA module colors. (j) Module-trait correlations heatmap displaying the relation between identified gene modules (rows) and experimental conditions. Colors represent correlation coefficients, Asterisks denote statistical significance (*p<0.05, **p<0.01, ***p<0.001). (k) Top enriched terms by WGCNA module showing functional annotation analysis. Different colored modules (blue, brown, dark turquoise, light yellow, pink, purple) are associated with specific biological processes (GO:BP), cellular components (GO:CC), molecular functions (GO:MF), and KEGG pathways. Circle size represents -Log (adjusted P-value).

To define transcriptional programs underlying lung pathology, lung tissue bulk RNA sequencing was performed across acute infection and Long-COVID phases (Fig. 2a). Principal component analysis (PCA) revealed distinct transcriptional clustering at 4 dpi, with lung samples from young males and young females separating from aged males, indicating sex- and age-dependent acute responses (Fig. 2b).

Differential expression analysis showed that most genes were group-specific with minimal overlap across age and sex (Fig. 2c). The common DEGs between 4 and 30 dpi were 195 in aged males, 279 in young males, and 80 in young females (Fig. 2d). The numbers of acute-phase (4 dpi) specific DEGs were 741, 1841, and 743 in aged males, young males, and young females, respectively, whereas Long-COVID (30 dpi) specific DEGs were 958, 512, and 290 (Figure 2d). KEGG pathway analysis of the shared DEGs identified enrichment of several neurological and cardiovascular pathways (Fig. 2e). Neurological pathways included dopaminergic synapses, neuroactive ligand-receptor interaction, calcium signaling, glutamatergic synapses, synaptic vesicle cycle, and HIF-1 signaling, whereas cardiovascular pathways included focal adhesion, ECM receptor interaction, vascular smooth muscle contraction, adrenergic signaling in cardiomyocytes, and cardiomyopathy-related pathways. Hierarchical clustering of genes within these pathways demonstrated clear sex- and age-stratified expression patterns, with young males exhibiting the most distinct profiles at 4 and 30 dpi (Fig. 2f and **Supplementary Fig. 2a-d**).

To assess immune cell composition in lung tissues, computational deconvolution of bulk transcriptomic data was performed using CIBERSORTx^29^. Monocytes, plasma cells, and naive B cells constituted the dominant immune populations across all groups. Activated NK cells were significantly enriched in young males at 30 dpi, whereas plasma cells were enriched in young females at both time points, and in young males during acute infection. Tfh cells were enriched in aged males and young females during acute infection, and in young males at 30 dpi. Interestingly, neutrophils and Tfh cells were both significantly increased in aged males during acute infection (Fig. 2g). Across groups, lung immune profiling revealed dominant monocytes, plasma cells, and naive B cells. Sex- and age-specific differences were represented by enriched plasma cells in young females, increased activated NK cells in young males (30 dpi), and elevated neutrophil and Tfh cell responses during acute infection, especially in aged males. Similar findings have been reported in human studies^30,31^.

To identify the transcriptional network associated with age, sex and infection phase, Weighted Gene Co-expression Network Analysis (WGCNA) was performed. A soft-thresholding power of 5 was used to achieve scale-free topology (Fig. 2h). Hierarchical clustering of gene co-expression identified initial (unmerged) modules, which were subsequently merged based on expression similarity to generate 24 distinct gene modules (Fig. 2i). Module-trait relationships are summarized in a correlation heatmap (Fig. 2j), where color intensity reflects the direction and strength of correlation (red, positive; blue, negative).

Several modules showed strong, condition-specific associations. The *brown* module was positively correlated with young males during acute infection (r=0.70, p<0.001) and negatively correlated with aged males at 30 dpi (r=−0.44, p<0.01). The *dark turquoise* module was positively associated with young females at 30 dpi (r=−0.62, p<0.001), while the *light-yellow module* correlated with young males during acute infection (r=−0.59, p<0.001). The *pink module* was associated with aged males at 30 dpi (r=−0.51, p<0.01). The *blue module* showed the strongest positive associations with acute-phase infection in both young males (r=0.44, p<0.01) and aged males (r=0.47, p<0.01), whereas the *purple module* was negatively associated with infection in young females (r=−0.45, p<0.01; Fig. 2 h–j).

Hub gene analysis revealed distinct trait-specific associations (**Supplementary Fig. 2e**). Metabolic genes, *Acadl* and *Acads* were positively associated with young male lungs, whereas immunoregulatory genes such as *Arid5a* and *Atf3* were negatively correlated, particularly in aged or acutely infected groups. Genes involved in immune cell trafficking and activation, such as *Ptk2b*, *Prkcq*, and *Rasal3* showed consistent associations across multiple traits, while *Ager* and *Nr4a1* genes, important for pulmonary immune homeostasis, were negatively correlated with inflammatory signatures.

Functional enrichment analysis of key modules showed distinct biological programs (Fig. 2k). The *blue module* was enriched for immune signaling pathways, including cytokine-mediated signaling, chemokine signaling, and B cell receptor signaling. The *brown module* was enriched for RNA-related processes and nuclear functions. The *dark turquoise module* was enriched for transcriptional regulation and stress-response pathways, including MAPK, and FoxO signaling. The *light-yellow module* showed enrichment for immuno-metabolic processes, including complement activity and lipid metabolism. The *pink module* was enriched for mitochondrial metabolic pathways, including the TCA cycle and fatty acid β-oxidation, while the *purple module* was enriched for extracellular matrix organization, receptor-mediated signaling, and extracellular matrix (ECM) remodeling.

Together, these analyses reveal pronounced sex- and age-dependent transcriptional and immune remodeling in SARS-CoV-2 infected lungs, with younger animals exhibiting dynamic immune activation and aged males displaying altered metabolic and immune regulatory networks.

### Sex-, age- and phase-dependent transcriptional and neuroimmune remodeling in SARS-CoV-2-infected brains

**Figure 3:**
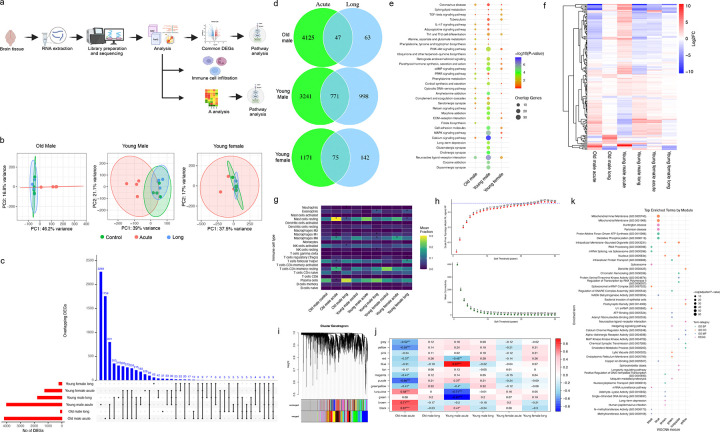
Sex- and age-dependent pathway enrichment, co-expression networks, and immune signatures in SARS-CoV-2–infected brains. (a) Schematic representation of brain transcriptomic analysis. (b) PCA plot showing sample clustering based on TMM-normalized gene expression. Ellipses represent group distributions for each group of animals across different conditions. The acute condition shows high variance in all the three groups. (c) Upset plot showing the overlap of differentially expressed genes (DEGs) among six experimental groups. Horizontal bars indicate total DEGs per group; vertical bars show the number of overlapping DEGs for each intersection. (d) Venn diagram showing overlap of DEGs between different experimental groups, with numbers indicating unique and shared DEGs across acute and Long-COVID. (e) Combined pathway analysis bubble plot showing significantly enriched pathways across different conditions (old male, young male, and young female). Bubble size represents the number of overlapping genes, and color intensity indicates -log10 (P-value). (f) Gene expression heatmap showing Log2 fold changes of DEGs identified across the pathways in different conditions and their status in other groups. (g) Immune cell fraction analysis showing the relative proportions of 22 different immune cell types across all experimental conditions compared to their respective controls, displayed as a heatmap with mean fraction values. (h) WGCNA soft threshold selection showing scale-free topology model fit (R^2^) and mean connectivity across different power values (1–30). The optimal soft threshold power was selected where R^2^ first exceeds the scale-free topology threshold while preserving network connectivity. (i) Gene clustering dendrogram showing WGCNA module colors. (j) Module-trait correlations heatmap displaying the relation between identified gene modules (rows) and experimental conditions. Colors represent correlation coefficients, Asterisks denote statistical significance (*p<0.05, **p<0.01, ***p<0.001). (k) Top enriched terms by WGCNA module showing functional annotation analysis. Different colored modules (blue, brown, dark turquoise, light yellow, pink, purple) are associated with specific biological processes (GO: BP), cellular components (GO: CC), molecular functions (GO: MF), and KEGG pathways. Circle size represents -Log (adjusted P-value).

Given the sex- and age-dependent transcriptional reprogramming observed in the lung and the high prevalence of neurological manifestations in COVID-19^32,33^, we next examined whether persistent molecular alterations occur in the brain following SARS-CoV-2 infection. Bulk RNA sequencing was performed on brain tissues across acute (4 dpi) and Long-COVID (30 dpi) phases, as outlined in Fig. 3a. The PCA revealed distinct clustering primarily in the young male acute group, whereas other groups exhibited comparatively limited transcriptional divergence (Fig. 3b). The UpSet plot demonstrated that most DEGs were unique to young and old during acute infection (Fig. 3c). The number of DEGs shared between 4 dpi and 30 dpi was 47 in aged males, 771 in young males, and 75 in young females (Fig. 3d). These patterns mirrored those observed in the lung.

KEGG pathway analysis of the DEGs common to both time points identified pathways related to neuroimmune signaling, synaptic regulation, and cellular signaling (Fig. 3e). These included TGF-beta, IL-17, adipocytokine, and Th1/Th2 signaling, as well as PI3K-Akt and cAMP signaling, serotonergic, glutamatergic, and dopaminergic synapses, calcium signaling, and ECM-receptor interactions. Hierarchical clustering of pathway-associated genes revealed clear sex- and age-stratified expression patterns, with young males exhibiting the most distinct transcription profiles at 4 and 30 dpi (Fig. 3f), while young females exhibited pronounced changes at 30 dpi only. Acute- and Long-COVID phase pathway enrichments further highlighted condition-dependent signaling and synaptic alterations (**Supplementary Fig. 3a-d**).

We next assessed immune cell composition in the brain using computational deconvolution of gene expression data. Macrophages (M0), plasma cells, and resting mast cells were consistently detected across groups (Fig. 3g). Macrophages were significantly enriched in aged males and young females at 4 dpi and in young males at 30 dpi. Resting mast cells were elevated in aged males at both time points, and in young females at 30 dpi, while eosinophils were significantly enriched in young females and males at the Long-COVID phase. Memory B cells were selectively enriched in young females at 30 dpi. Overall, the young animals exhibited greater immune cell infiltration at the Long-COVID phase.

To define coordinated transcriptional programs, WGCNA was performed, and a soft-thresholding power of β = 26 was selected to achieve a scale-free topology with a high model fit (R^2^>0.8; Fig. 3h and 3i). Thirteen co-expression modules were identified and merged based on eigengene similarity (Fig. 3j). Module–trait correlation analysis revealed strong, condition-specific associations. The *brown module* was positively correlated with aged males during acute infection (r=0.71, *p*<0.001), while the *yellow module* was negatively correlated (r=−0.59, *p*<0.001). The *black modules* were also positively associated with acutely infected young males (r=0.67, *p*<0.001). In contrast, the *turquoise module* showed contrasting correlations with young males (negative, r=−0.71, *p*<0.001) and aged males (positive, r=0.58, *p*<0.001) during acute infection (Fig. 3j). The *blue and green modules exhibited* the strong positive (r=0.87, *p*<0.001) and negative (r=−0.92, *p*<0.001) correlations, respectively, with young males during acute infection, underscoring pronounced age- and phase-dependent transcriptional organization.

Hub gene analysis identified brain-specific regulatory signatures (**Supplementary Fig. 3e**). Lrch4 showed a strong positive association with the young male during acute infection (r=0.6, p<0.001), while Fam193b and Rpl35 showed moderate correlations (r=0.4 and 0.2, respectively). Ptk2b was negatively correlated with multiple traits across conditions (r=−0.4), and immune signaling genes, including Prkcq, and Rasal3, also showed trait-specific associations, particularly during acute infection among aged male GSHs. Notably, synaptic and neuronal genes (Syn1, Mapk8ip2, and Sptbn2) were associated with the Long-COVID phase in both young and aged males, suggesting the presence of age-agonistic gene regulatory networks in male GSHs.

Functional enrichment of key WGCNA modules revealed distinct neurobiological processes (Fig. 3k). The *black module* was enriched for RNA processing and splicing, including the spliceosome, and mRNA surveillance pathways. The *blue module* was enriched for mitochondrial processes, oxidative phosphorylation, and neurodegenerative disease pathways, including Parkinson’s disease (PD) and Huntington’s disease (HD). The *brown module* was enriched for nuclear transport, transcriptional regulation, and DNA binding functions. The *green module* was enriched for intracellular trafficking, vesicle dynamics, and protein turnover. The *turquoise module* was enriched for chromatin remodeling, transcriptional regulation, kinase activities, and aging-related neurodegenerative pathways. The *yellow module* was enriched for synaptic signaling, neuroactive ligand–receptor interaction, dendritic functions, and cholesterol metabolism, highlighting neuroimmune and neuronal regulatory roles.

Collectively, these data demonstrate that SARS-CoV-2 infection induces pronounced sex-, age- and disease phase-specific transcriptional and neuroimmune remodeling in the brain, characterized by distinct gene co-expression modules and pathway enrichments that may underlie persistent neurological manifestations associated with Long-COVID.

### Sex- and age-dependent perturbation of neurotransmitter pathways after SARS-CoV-2 infection

**Figure 4:**
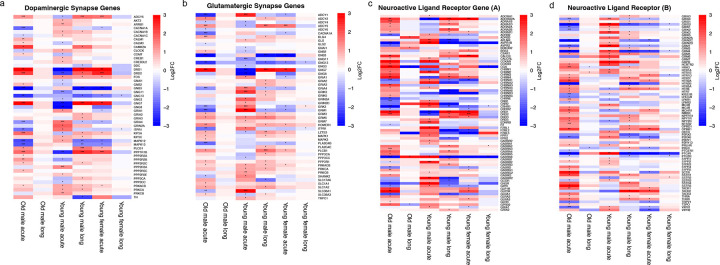
SARS-CoV-2 infection disrupts synaptic signaling and neuroactive ligand-receptor pathways, with young male showing the greatest transcriptional alterations during Long-COVID. (a) Heatmap showing the differentially expressed genes at 4 and 30 dpi according to the selected KEGG pathway “Dopaminergic synapse” (b) “Glutamatergic synapse”, and (c-d) “Neuroactive-ligand receptor” calculated in comparison with mock-infected hamsters. Color gradient represents the transcription log2 fold change comparing infected and mock-infected derived from edgeR differential expression analysis with TMM normalization. Statistical significance in heatmaps is indicated by asterisks based on raw p-values: * (p < 0.05), ** (p < 0.001), *** (p < 0.0001).

DGE analysis revealed significant disruption of multiple neurotransmitter systems following SARS-CoV-2 infection, with the most pronounced alterations observed in young male hamsters. Within the dopaminergic pathway, at acute infection, downregulation of dopamine receptors (DRD1, DRD2) were observed in young male GSHs, whereas these genes were upregulated in young female, and old GSH during acute infection (4 dpi) and in young males during Long-COVID phase (30 dpi) (Fig. 4a), indicating sex- and age-dependent differential regulation at different stages of the disease.

The glutamatergic signaling pathway was also persistently altered, with changes detected in AMPA receptor genes (GRIA1–4), NMDA receptor subunits (GRIN2A, GRIN2D), and metabotropic glutamate receptors (GRM1, GRM3, GRM5) across infection phases (Fig. 4b). Analysis of the broader neuroactive ligand–receptor interaction pathway identified 140 DEGs, encompassing genes associated with GABAergic, serotonergic (HTR1A, HTR2A), adrenergic, and neuropeptide receptors (Fig. 4c and 4d). Consistent with these findings, pathways associated with neurodegenerative diseases, including AD and PD were significantly enriched, with the strongest transcriptional perturbations observed in younger males during acute infection (**Supplementary Fig. 4a–c**). Altogether, these data demonstrate sustained sex- and age-dependent disruption of core neurotransmitter signaling networks following SARS-CoV-2 infection.

### Sex- and age-dependent transcriptomic remodeling across lungs, heart, and brain during Long-COVID

To assess organ-specific transcriptional remodeling during the Long-COVID phase, we performed comparative transcriptomic analyses of lung, heart, and brain tissues from aged and young male GSHs. In aged males, each organ displayed both distinct and overlapping gene expression changes, with the lung exhibiting the highest number of unique DEGs (**Supplementary Fig. 5a**). Enrichment analyses revealed shared and tissue-specific pathways related to immune regulation, metabolic signaling, and neurodegeneration-associated processes (**Supplementary Fig. 5b, 5c, 5e and 5f**). Heatmap analysis showed tissue-specific transcriptional signatures, as only three genes were differentially expressed across the three different tissues (**Supplementary Fig. 5d**). FGG, DBP and SGK1 were common genes differentially expressed across the three tissues and are linked with coagulation, circadian transcriptional control, and hormone-responsive cell signaling^34–36^.

In young males, comparative analysis likewise revealed extensive organ-specific and overlapping transcriptional changes (**Supplementary Fig. 6a**). Notably, the brain exhibited the largest number of unique DEGs, and pathway enrichment spanned metabolic processes, immune signaling, and neurodegeneration-related pathways (**Supplementary Fig. 6b, 6c, 6e and 6f**). Heatmap analysis of commonly altered genes demonstrated predominant downregulation across all three tissues, suggesting coordinated suppression of shared transcriptional programs during the Long-COVID phase (**Supplementary Fig. 6d**).

### Sex- and age-dependent behavioral deficits and brain microstructural remodeling following SARS-CoV-2 infection

**Figure 5:**
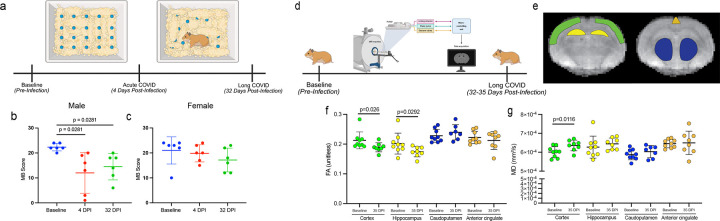
Sex-dependent behavioral and brain microstructural changes in hamsters following SARS-CoV-2 infection. (a) Schematic representation of behavioral assessment. (b) Marble burying score of male animals. (c) Marble burying score of female animals. Statistical analysis by the Friedman test with Dunn’s post-hoc comparisons was used to compare between timepoints (d). Schematic representation of DT-MRI analysis at baseline and 35 dpi. (e) Representative MRI image overlays showing corresponding brain regions used for DTI quantification. (f) Fractional anisotropy (FA) values measured by in vivo diffusion tensor imaging (DTI) in cortex, hippocampus, caudoputamen, and anterior cingulate regions at baseline and 35 DPI. Significant reductions in FA were observed in the cortex and hippocampus. (g) Mean diffusivity (MD) values in the same regions showing significant increase in all the regions at 35 dpi. Statistical significance was assessed using unpaired t-tests.

To assess behavioral consequences of SARS-CoV-2 infection, compulsive-like behavior was evaluated using the Marble burying (MB) test (Fig. 5a). In aged males, MB scores were significantly reduced at 4 dpi compared with baseline (*p*=0.0281) (Fig. 5b), which persisted at 30 dpi (*p*=0.0281), indicating sustained behavioral alteration following SARS-CoV-2 infection. In contrast, females showed no significant changes across the baseline, 4 dpi, and 32 dpi (Fig. 5c).

Given the persistent transcriptional and behavioral changes, we next examined brain microstructural alterations using diffusion tensor magnetic resonance imaging (DT-MRI) in a subset of animals (Fig. 5d). Analysis focused on brain regions implicated in cognition and memory, including the cortex, hippocampus, caudoputamen, and anterior cingulate area (Fig. 5e). Across all animals, fractional anisotropy (FA) an index of white matter microstructural integrity, was significantly reduced (*p* <0.05) in the cerebral cortex and hippocampus at 30 dpi compared with baseline (Fig. 5f), whereas Mean diffusivity (MD) an index of diffusional freedom in a tissue, was significantly increased in the cerebral cortex (Fig. 5g).

Taken together, these results indicate that SARS-CoV-2 infection induces sex-specific behavioral changes and has a compromised microstructural organization in brain regions critical for cognition and memory. Alterations in FA and MD are consistent with disrupted tissue organization and altered cellular microenvironments, providing a structural correlate for the sustained neurological and cognitive impairments associated with Long-COVID.

### Sex- and age-dependent dysregulationtions in α-synuclein homeostasis and microglial activation after SARS-CoV-2 infection

To assess SARS-CoV-2-mediated microglial activation and α-synuclein (α-syn) pathology, immunofluorescence staining for IBA-1 and α-syn was performed on brain sections in a subset of GSHs (**Supplementary Fig. 7a**). Analysis focused on the hippocampus, a region vulnerable to α-syn pathology in neurodegenerative disorders, including PD^37^. Compared with controls, SARS-CoV-2–infected animals exhibited increased α-syn immunoreactivity within the hippocampus (**Supplementary Fig. 7b and 7c**).

Quantitative analysis revealed increased density of IBA1-positive microglia (cells per mm^2^) in SARS-CoV-2-infected brains compared to controls (**Supplementary Fig. 7d**). However, the proportion of morphologically activated microglia was reduced in infected animals (**Supplementary Fig. 7e**). Notably, activated microglia in infected brains displayed a significantly greater average process area per cell (**Supplementary Fig. 7f**) and thicker, hypertrophic processes consistent with altered activation (**Supplementary Fig. 7g**).

To further examine α-syn homeostasis, we assessed phosphorylation at serine 129 (pS129), a modification critical for α-syn function and synaptic regulation. Overall, pS129 levels were significantly reduced in SARS-CoV-2-infected brains compared to controls. The aged male GSHs showed lower baseline levels of pS129 compared to young males (~0.8-fold, p=0.0328) (**Supplementary Fig. 7h**). Further, young males exhibited a reduction in pS129 at 30 dpi (*p*=0.0225). Together, these findings demonstrate that SARS-CoV-2 infection disrupts α-synuclein homeostasis and alters microglial activation states in a sex- and age-dependent manner. Sustained suppression of pS129 in the Long-COVID phase suggests persistent perturbation of synaptic and neuroimmune regulatory mechanisms, linking SARS-CoV-2 infection to long-term neurobiological alterations.

### Sex- and age-dependent alterations in gut microbiota composition following SARS-CoV-2 infection

**Figure 6:**
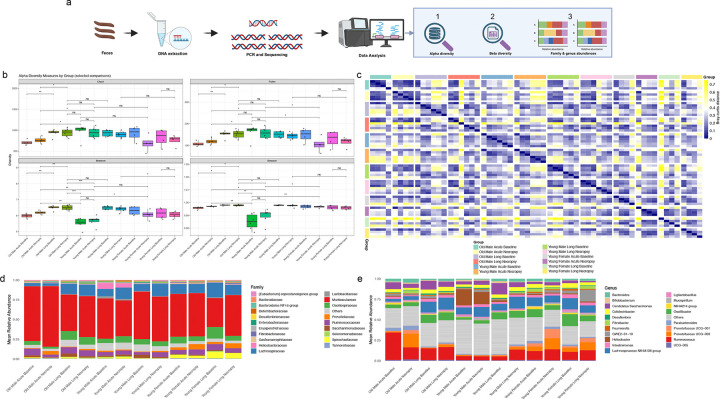
Sex- and age-specific gut microbiome analysis of acute and Long-COVID hamsters. (A) Schematic representation of microbiome analysis. (B) Box plot representing the alpha diversity indices, indicating microbial richness and evenness across different sample groups, suggesting potential alterations in gut microbial composition associated with disease state. (C) Beta-diversity analysis of the fecal microbiome across sample groups. Pairwise distances between samples were calculated using the Bray–Curtis dissimilarity method and visualized as a heatmap. Each row and column corresponds to an individual sample, with the color intensity representing the degree of similarity or dissimilarity in microbial community composition. Clustering patterns highlight microbial communities from different groups, providing insights into group-specific microbial shifts. Mean relative abundance of microbial profiles showing altered gut microbiota across different groups at the (D) family and (E) genus level. The top 20 most abundant taxa were identified and compared between groups at both the family and genus levels, respectively. Each bar represents the average community composition within a group, with different colors corresponding to distinct microbial families or genera, showing shifts in top taxa that differentiate different infection groups.

We previously demonstrated that SARS-CoV-2 (Omicron) infection induces acute alterations in gut microbiota composition in GSHs^27^. Given reports of post-COVID gastrointestinal and gut–brain axis (GBA) dysfunction^38,39^, we assessed fecal microbiota composition at both acute (4 dpi) and Long-COVID (30 dpi) stratified by age and sex (Fig. 6a). Alpha diversity analysis (Chao1, Fisher, Shannon, and Simpson indices) revealed no significant differences between baseline and 4 dpi when assessed globally; however, significant sex- and age- specific differences emerged across groups (Fig. 6b). In male groups, both young and aged animals exhibited higher microbial richness and diversity metrics at baseline and 4 dpi compared with their 30 dpi counterparts, indicating a loss of microbial richness following infection. Chao1 and Fisher indices showed reduced richness (*p<0.05 and **p<0.001), while Shannon and Simpson indices showed reduced evenness and diversity in necropsy samples, with more pronounced effects observed in young males (*p<0.05 and **p<0.001). In contrast, female groups exhibited relatively stable or partially recovered diversity levels, profiles over time consisted with microbial re-equilibration. Together, these data indicate that infection, age, and sex together influence microbiome diversity, with males showing the greatest loss of richness and uniformity.

Beta diversity was next assessed using Bray-Curtis dissimilarity to evaluate compositional differences between groups (Fig. 6c). Aged males at 4 dpi exhibited moderate dissimilarity relative to baseline, indicating acute-phase microbial structuring. In contrast, aged males at 30 dpi showed greater dissimilarity, reflecting substantial alterations in microbial composition. Young males at 30 dpi displayed lower dissimilarity between baseline and necropsy samples compared with aged males, suggesting comparatively greater microbial stability during disease progression.

Taxonomic profiling revealed pronounced sex- and age-specific differences were evident at family and genus levels (Fig. 6d and 6e). At the family level, *Muribaculaceae* was abundant across all groups but enriched in aged males, while *Lachnospiraceae* was reduced during acute infection. *Helicobacteraceae* was selectively enriched in young males, particularly at the acute stage, whereas Spirochaetaceae was enriched in young females, with the highest abundance observed at 30 dpi.

At the genus level, aged males exhibited broadly similar microbial compositions across baseline, 4 dpi, and 30 dpi, although *Ruminococcus* was more abundant at the baseline during acute infection. In contrast, *Helicobacter* was markedly enriched in young males at both baseline and necropsy during acute infection, but remained lower in all other groups, suggesting a cohort-specific response. Additionally, *Candidatus Saccharimonas* was relatively abundant across most groups but declined markedly by necropsy. In females, *Prevotellaceae UCG-001* and *GWE2–31-10* were enriched, with *GWE2–31-10* showing the highest abundance in young females at the 30 dpi. Collectively, these results demonstrate that SARS-CoV-2 infection induces sex- and age-dependent gut microbiota dysbiosis that evolves across disease stages.

At the genus level, dbRDA axis 1 coefficient analysis revealed positive correlation of some bacteria, such as *Prevotellacea* UCG-003, *Lachnospiraceae* NK4A136 group, *Ruminococcus*, and GEW2–31-10, indicating higher abundance of these taxa across some groups. In contrast, genera such as *Ruminococcus*, *Helicobacter*, *Candidatus Saccharimonas*, and *Oscillibacter* showed negative coefficients (**Supplementary Fig. 8b**). Notably, changes in some of these taxa, such as *Ruminococcus* and *Candidatus Saccharimonas*, have been previously reported to modulate inflammatory genes, cell antimicrobial level, inflammatory bowel diseases, or cognitive performance. Thus, a decrease in abundance suggests potential relevance to SARS-CoV-2 infection-associated gut dysbiosis and neurological dysfunction. At the family level, Muribaculaceae, Lachnospiraceae, and Prevotellaceae were positively correlated (high abundance), whereas families like Ruminococcaceae, Saccharimonadaceae, and Oscillospiraceae showed low abundance in some groups (**Supplementary Fig. 8a**). These variations differ across different age groups and sexes. The pronounced alterations observed in male animals, particularly at the genus level, suggest persistent disruption of microbial community structure that may contribute to differences in recovery trajectories and systemic outcomes following SARS-CoV-2 infection.

## Discussion

Long-COVID encompasses a broad spectrum of persistent symptoms lasting weeks to months after the resolution of the acute phase and affects millions of individuals worldwide^15,40^. These symptoms span multiple organs, including the respiratory, cardiovascular, gastrointestinal, and nervous systems, yet the underlying molecular mechanisms remain incompletely defined. Emerging evidence suggests that unresolved inflammation, immune dysregulation, microbiota disruption, and/or viral persistence contribute to sustained pathology. In this study, we employed an integrated systems-level approach to understand how SARS-CoV-2 infection drives sex-, age- and organ-specific pathologies, with a particular focus on neurological outcomes that represent a major burden for people experiencing Long-COVID.

The GSH represents a robust and translationally relevant model to study SARS-CoV-2 pathophysiology and post-acute sequelae^26,27^. Unlike murine models that require genetic modification to permit viral entry, GSH naturally expresses ACE2 compatible with SARS-CoV-2 infection^41^, closely recapitulating key aspects of human disease, including the respiratory pathology, cytokine responses, and multi-organ involvement^26,28,41^. Our findings extend prior work by demonstrating that model also captures salient features of Long-COVID by exhibiting persistent viral RNA, long-lasting transcriptional reprogramming in lungs, heart, and brain, and sustained behavioral alterations resembling neuropsychiatric symptoms reported in humans^42,43^. Importantly, the model revealed pronounced sex- and age-dependent differences in host responses mirroring demographic disparities observed in Long-COVID patients and underscoring its utility as a preclinical model in investigations of individualized therapeutic strategies.

Persistent symptoms following SARS-CoV-2 infection have been reported for up to two years in some individuals^44,45^, and several non-mutually exclusive mechanisms have been proposed, including viral persistence, immune dysregulation, reactivation or acceleration of underlying neurological disease, and gut-brain axis dysfunction^46^. Accumulating evidence indicates that viral RNA or antigen can persist in tissues long after resolution of acute infection, sustaining local immune activation despite the absence of replication-competent virus^47,48^. Consistent with recent human autopsy studies identifying SARS-CoV-2 RNA in multiple organs months after infection^40,49,50^, we observed persistence of viral RNA in several tissues/organs of SARS-CoV-2-infected GSHs. Notably, viral RNA remained detectable in the lungs of a subset of male GSHs at 30 dpi. This sex-biased persistence parallels clinical observations of greater disease severity and poorer outcomes in male COVID-19 patients^51,52^.

Sustained viral RNA was accompanied by persistent host transcriptional alterations extending beyond the acute phase. Transcriptomic profiling revealed striking sex- and age-specific effects, particularly in the brain. Young males exhibited the largest numbers of DEGs shared between acute and post-acute phases, indicating prolonged disruption of neural gene regulatory networks. While overlapping pathways were identified in the brain, lung, and heart, including immune and signaling cascades, the expression patterns were organ-specific, suggesting differential tissue susceptibility and adaptation. Notably, neurological pathways, such as dopaminergic and glutamatergic synapses and neuroactive ligand–receptor interactions, were enriched not only in the brain but also in lung and heart transcriptomes, raising the possibility that peripheral transcriptomic signatures may serve as accessible biomarkers of central nervous system perturbations.

Enrichment of pathways related to neuroinflammation, synaptic signaling, and TGF-beta signaling directs to fundamental disruptions in neural communication and plasticity^53–55^. Neurotransmitter systems pathways governing mood, cognition, and motor function, including serotonergic, glutamatergic, and dopaminergic signaling, were particularly affected, aligning with clinical reports of brain fog, depression, anxiety, and cognitive impairment in Long-COVID patients, especially among younger males^45,56,57^. Sex- and age-dependent clustering supports epidemiological data indicating that Long-COVID risk and symptoms vary by demographic factors^58,59^. The comparatively blunted transcriptional responses in aged males may reflect immunosenescence and reduced adaptive capacity, potentially contributing to higher mortality during acute infection observed in this group^60,61^. Clinical studies also reported that younger males are at greater risk for neurologic complications in Long-COVID^62^.

Behavioral analyses reinforce these molecular findings. Reduced MB test score, particularly in males, suggests persistent alterations in compulsive-like and goal-directed behaviors following infection. While a lower MB score is often interpreted as a measure of anxiety and compulsive behavior, the study outcomes may also reflect apathy-like phenotypes, a common neuropsychiatric feature of Long-COVID^63,64^. The sex-specific differences of these deficits align with emerging clinical evidence that neurobehavioral sequelae of COVID-19 differ between males and females^65^.

Advanced neuroimaging further demonstrated that SARS-CoV-2 infection induces long-term microstructural changes in the brain^66,67^. Reductions in FA and increases in MD in the cerebral cortex and hippocampus indicate compromised tissue organization and altered cellular microenvironments^68^. These brain regions are critical for cognition, memory, motivation, and emotional regulation^69–71^, and are particularly vulnerable to hypoxia, inflammation, and stress^72,73^. In our study, the persistence of these changes at 30 dpi as compare to baseline suggests lasting structural remodeling rather than transient inflammation and parallels diffusion tensor MRI abnormalities reported in human Long-COVID cohorts^63,64,74–76^. Importantly, these imaging findings are concordant with transcriptomic alterations affecting synaptic and signaling pathways, providing convergent molecular and structural evidence of sustained neurological impact. We further observed increased microglial density and altered activation states, together with disruptions of α-synuclein homeostasis in the hippocampus. Accumulation and dysregulation of α-synuclein, a hallmark of PD and related synucleinopathies, combined with sustained neuroinflammation, raises the possibility that SARS-CoV-2 infection may accelerate or unmask neurodegenerative processes in susceptible individuals^77–79^. These findings are consistent with post-mortem studies of COVID-19 patients reporting microglial activation and neuroinflammatory changes and align with prior evidence that respiratory viral infections can trigger central nervous system inflammation and neurodegeneration via direct invasion, systemic inflammation, or immune-mediated mechanisms^80,81^. Although some studies have reported increased microglia density, our study, however, supports studies that showed a similar number of microglia in the hippocampus^82,83^. This finding further corroborates the evidence of post-mortem studies of COVID-19 patients showing microglial activation and neuroinflammatory changes^84,85^.

In parallel, our microbiome analyses highlight a potential role for the GBA in shaping long-term neurological outcomes. Emerging evidence indicate at significant role of the GBA in modulating COVID-19-related neurological outcomes, especially in Long-COVID, by disturbing the gut microbiota homeostasis, resulting in systemic and neuroinflammation^86,87^. SARS-CoV-2 infection induced marked sex- and age-dependent gut microbiota dysbiosis, particularly at the genus level. This aligns with prior reports where sex hormone- and aging-dependent alterations in gut microbiome composition and immune responses are associated with infection and inflammation susceptibility^88,89^. Beta diversity analyses revealed clear segregation by disease status, age, and sex, supporting the concept that host biology strongly modulates infection-induced dysbiosis^90,91^. Notably, reductions in taxa such as *Ruminococcus* and *Candidatus Saccharimonas,* which are implicated in anti-inflammatory functions and gut homeostasis, may exacerbate systemic inflammation and contribute to GBA dysfunction^92^. These microbial signatures may represent potential microbial biomarkers or therapeutic targets for mitigating neurological sequelae of COVID-19.

**Figure 7:**
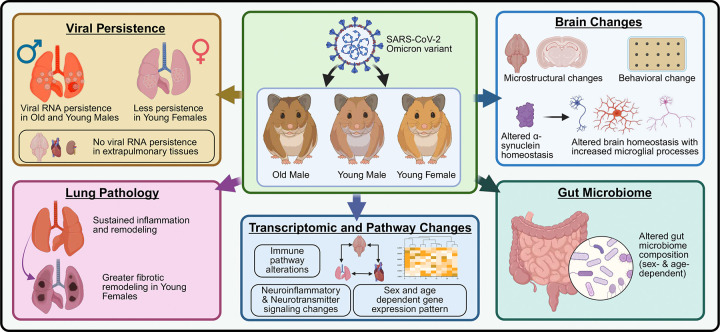
Multi-system alterations associated with long COVID following SARS-CoV-2 infection in hamsters. Graphical summary of the data presented in this manuscript. In brief, age- and sex-dependent changes seen in GSHs (old males, young males, and young females) throughout the post-acute phase of SARS-CoV-2 infection. Both young and old males had viral RNA persistence in their lungs, but young females had less viral persistence; extrapulmonary organs showed no viral RNA persistence. Lung pathology showed persistent tissue remodeling and inflammation, with young females showing more fibrotic remodeling. Brain investigations revealed behavioral and microstructural changes, along with enhanced microglial activities and impaired α-synuclein homeostasis, indicating altered brain homeostasis. Changes in immunological pathways, neuroinflammatory and neurotransmitter signaling, and sex- and age-dependent gene expression patterns were observed using transcriptomic profiling. Concurrently, there were age- and sex-dependent changes in the makeup of the gut microbiome. Together, these findings highlight multi-organ changes associated with Long-COVID that vary according to host sex and age.

Taken together, our findings demonstrate that SARS-CoV-2 infection allows a long-lasting, non-uniform molecular and functional imprint across multiple organ systems, strongly shaped by host age and sex (Fig. 7). In our hamster model, young males exhibited the most pronounced and persistent neuroimmune and transcriptomic alterations, while young females showed greater fibrotic remodeling in the lung. These molecular changes were reflected in behavioral deficits, neuroimaging abnormalities, and gut microbiota dysbiosis, providing an integrated framework for understanding the heterogeneity of Long-COVID. Importantly, these results underscore the value of preclinical models such as the Golden Syrian hamster for dissecting demographic influences on Long-COVID and for guiding the development of personalized therapeutic strategies.

## Methods

### Virus

SARS-CoV-2 strain hCoV-19/USA/GA-EHC-2811C/2021 (Lineage B.1.1.529; Omicron Variant; # NR-56481) was obtained from BEI resources. The virus was propagated on Calu-3 cells maintained in Eagle’s Minimum Essential Medium (ATCC30–2003) supplemented with 10% fetal bovine serum (FBS), 2 mM L-glutamine, 100 U/ml of penicillin, and 100 μg/ml streptomycin. The titers were determined using a plaque assay performed on Vero E6 cells, as previously described^93^. For all animal experiments, virus stock derived from passage 1 or 2 in Calu-3 cells was used.

### Animal experiments

Golden Syrian Hamsters (GSHs, from Charles River Laboratories) were stratified into young (8–16 weeks), and aged (36–40 weeks) cohorts. The experimental schema is outlined in Fig. 1a. The study included young male (n=18; six each in control, acute infection, and Long-COVID group), young female (n=18; six each in control, acute infection, and Long-COVID group), and aged male hamsters (n=24; six each in control and acute infection group, and 12 in Long-COVID group). Animals were randomly assigned to each group to minimize selection bias. All animal procedures were approved by UNMC IBC (20–05-029) and IACUC (21–019-06), and the SARS-CoV-2-related work was conducted in the ABSL-3/BSL-3 facility.

GSHs were intranasally infected with the SARS-CoV-2 (16,000 PFU in 100μl) following anesthesia with isoflurane as previously described by our lab^26,27^. The uninfected control groups received PBS. The control and acute COVID-19 group GSHs were euthanized using ketamine and xylazine injection at 4 days post-infection (dpi), while the Long-COVID group animals were euthanized at 30–35 dpi (referred to as 30 dpi or Long-COVID hereafter).

At necropsy, trans cardiac perfusion was performed with 60 ml ice-cold PBS injected into the heart under constant pressure until venous outflow was observed^94^. After perfusion, tissues from the brain, heart, kidney, lung, and small intestine were collected. The blood and feces were collected at day 0 and Day 4 post infection (for Control and Acute), and on day 0 and 32 – 35 (for the Long-COVID group, referred to as 30 dpi or long hereafter). Simultaneously, the oral swabs were collected at day 0, 2, and 4 (control and acute) and on day 0, 2,4,11, and 27 post infection (for 30 dpi or Long-COVID group).

### SARS-CoV-2 viral genomic RNA quantification

RNA from oral swabs, plasma, and fecal samples was isolated using the QIAamp Viral RNA Mini Kit (Qiagen; Cat#52906) according to manufacturer specifications. Flash-frozen tissues from GSHs were homogenized in RLT buffer using TissueLyser LT (Qiagen). Subsequently, total RNA was extracted using the RNeasy Mini Kit (Qiagen; Cat#74106) following the manufacturer’s instructions. One-step RT-qPCR was performed to quantify viral genomic (E gene) from oral swabs using specific primer-probes and QuantStudio3 real-time PCR system (Applied Biosystems) per manufacturer’s specifications^27^. Quantification of SARS-CoV-2 genomic (E gene) RNA in tissues, feces, and plasma was carried out by RT-ddPCR using Bio-Rad QX200 AutoDG system, gene-specific primers, and probes previously described by our group^27^. Briefly, the total reaction volume for each sample was 22 μl, and it contained 5 μl 4X Supermix, 2 μl 10X reverse transcriptase enzyme, 500 nM of each primer, 250 nM probes, and 10 μl of RNA template. Droplets were generated, and the emulsified samples were sealed, and amplification was performed using the C1000 Touch thermal cycler under the following conditions: 48 °C for 60 min, 95 °C for 10 min, followed by 50 cycles of 95°C for 30 sec and 55°C for 1 min, and a final extension at 98 °C for 10 min. Droplet fluorescence was read with the QX200 droplet reader.

### Histopathological Analysis

For Hematoxylin and Eosin (H&E) staining, lung tissues were fixed in 10% neutral phosphate-buffered formalin for 3 days. Next, tissues were placed in cassettes and processed in STP 120 (Thermo Scientific) tissue processor using a graded series of ethanol, xylene, and paraffin wax. Paraffin-embedded tissue blocks were sectioned at a thickness of 5 μm and subjected to standard H&E staining for histological evaluation. Images were captured using the Echo Revolution microscope at 4x and 10x magnification. A board-certified pathologist, blinded to the groups, assessed the stained slides using a scoring system: 0 = no pathology; 1 = minimal pathology; 2 = mild to moderate pathology; 3 = severe pathology; 4 = hyperplasia.

Next, to quantify collagen deposition in the lung tissues, picrosirius red staining was performed. Slides were baked for 1 hour to remove paraffin wax and deparaffinized in xylene. Sections were rehydrated through descending grades of alcohol, followed by a gentle rinse under tap water. Sections were then stained with Picrosirius red stain, washed in 0.5% acetic acid, through ascending grades of alcohol, cleared in xylene, and mounted with Cytoseal. Images were captured using the Echo Revolution microscope (Discover Echo Inc; Model RON-K) at 4x and 10x magnification. Collagen deposition was quantified using ImageJ software and expressed as a percentage of total tissue area across the whole tissue section.

### Sample preparation, RNA sequencing, and functional enrichment analysis

Total RNA (1 μg) from each lung, brain, and heart sample (**Supplementary Tables 1 and 2**) was used to prepare the sequencing libraries with NEB Ultra II Directional library Prep kit with Poly-A Selection, following the manufacturer’s instructions. Libraries were sequenced using the Illumina NovaSeq 6000 platform, generating 150 bp paired-end reads. The adapter-trimmed FASTQ files were aligned to the *Mesocricetus Auratus* reference genome (Mesocricetus_auratus.MesAur1.0.111.gtf, Ensembl) using the STAR (v2.7.9a). Gene counts were generated with FeatureCounts (Subreads, v2.0) and analyzed using the edgeR pipeline (v3.40.2) in R (v4.2.2). Low-abundance genes (<10 counts in at least two samples per group) were filtered out. The remaining count data were normalized using the Trimmed Mean of M-values (TMM) method via the *calcNormFactors* function. Biological dispersion was estimated using *estimateCommonDisp* and *estimateTagwiseDisp.* Differential gene expression analysis between groups was determined using the *exactTest* function in edgeR using the normalized counts. The resulting p-values were corrected for multiple testing using the Benjamini-Hochberg (BH) procedure, and genes with p-value<0.05 and a log2FC of |≥0.6| were considered significantly differentially expressed.

Principal component analysis (PCA) was performed using edgeR, ggplot2, ggforce, and tidyverse, and plots were generated. Pathway analysis was performed using enrichR (v3.2) in the R environment as described elsewhere^95^. Additional visualization and R packages included MASS, readr, calibrate, and VennDiagram for Venn diagram generation, ggplot2, dplyr, and readr for bubble plot generation.

### Immune cell infiltration analysis

Immune cell infiltration analysis was performed using the CIBERSORTX online platform with the LM22 signature matrix to estimate immune cell composition as described previously^29^. A mixture file was prepared with row names as gene symbols and columns as samples with expression values. GSHs gene names were converted to human gene symbols using the biomaRt package in R as described earlier^96^. For RNA data, quantile normalization was disabled, and analysis was run with 1000 permutations for the brain and lung sample datasets independently. The resulting immune cell fraction matrix was integrated with sample metadata and reshaped for downstream analysis. Differential abundance of immune cell types for each condition was assessed using the Wilcoxon rank-sum. Mean immune cell fractions were calculated for each condition, and significance levels were annotated based on p-values (***p≤0.001, **p≤0.01, *p≤0.05). Control groups for Wilcoxon analyses were defined through age and sex matching, where each treatment condition (acute/Long-COVID) was compared exclusively to its corresponding control group from the same age and sex category (young male, old male, and young female). A heatmap was generated using ggplot2, with color representing mean cell fraction and asterisks denoting statistical significance.

### Weighted gene co-expression network analysis (WGCNA)

For the WGCNA analysis, normalized RNA-Seq count data were processed using *DESeq2* in R as described previously^97^. Genes with low expression (fewer than 15 counts in over 75% of samples) were filtered out, and a variance stabilizing transformation (VST) was applied to obtain normalized expression values. WGCNA was performed using the VST-transformed data to identify co-expression modules as described earlier^98^. Soft-thresholding powers of 5 (lung) and 26 (brain) were chosen based on scale-free topology criteria. Gene modules were identified using the *blockwiseModules* function, and module eigengenes were correlated with phenotypic traits to identify biologically relevant modules. Significant module-trait associations were visualized using *CorLevelPlot*, and genes from selected modules were extracted for functional enrichment and downstream analysis.

To identify key regulatory genes within biologically relevant modules, we performed hub gene analysis using WGCNA. Modules of interest were selected based on their eigengene-trait correlations, and the top 5 hub genes from each of six modules were extracted based on their module membership (kME) values. These hub genes represent the most centrally connected genes within their respective modules and are likely to play critical roles in the underlying biological processes. We further examined the relationship between these hub genes and external phenotypic traits by calculating gene significance (GS) values, defined as the Pearson correlation between individual gene expression profiles and trait measurements. A heatmap of gene-trait correlations was generated, annotated with significance levels (***p≤0.001, **p≤0.01, *p≤0.05), highlighting genes with strong associations to key traits.

### Functional enrichment of WGCNA modules

Functional enrichment analysis was performed on gene modules identified from WGCNA of brain (Black, Blue, Brown, Green, Turquoise, and Yellow) and lung (Blue, Brown, Dark Turquoise, Light Yellow, Pink, and Purple) samples using the Enrichr online tool as described previously^95^. GO enrichment results for each module were exported as CSV files, filtered, and merged into a single dataset. The top two GO terms per module were selected based on the highest LogP values. A dot plot was generated using ggplot2 to visualize enriched GO terms across modules, with point size representing the enrichment significance.

### Marble burying assay

The marble burying assay (MBA) was adapted from previously described protocols^99^. We performed the MBA at baseline, at four dpi (model acute infection), and at 30 dpi (model PASC). A total of 12 animals were included in this study (50% of both sexes). This is a well-validated task that enables us to evaluate obsessive-compulsive behaviors, autism spectrum disorders (ASD), and anxiety-like behaviors in pre-clinical animal models, such as rodents^100–102^. The Marble Burying Scoring was assigned as: 2 points for each fully buried marble, 1 point for each half-buried marble and partially buried marble, and zero points for each marble not buried.

### Radiological Investigations

Diffusion tensor magnetic resonance imaging (DT-MRI) was conducted at baseline and 30 dpi using a 7 Tesla Bruker preclinical MRI scanner. In brief, GSHs were anesthetized with 1.5%–2.5% isoflurane delivered with oxygen at a flow rate of 1 L/minute. Imaging parameters included: slices=15, slice thickness=0.5mm, image matrix=128×128, signal averages=1, repetitions=7, b-value=800 s/mm^2^, diffusion directions=12, segments=4, and scan duration =12m08s. In-house developed software was used to remove distorted images, and fractional anisotropy (FA) and mean diffusivity (MD) maps were generated using Diffusion Toolkit software^103^. Also, because of the unavailability of a labelled MRI-based hamster brain atlas, the Allen-mouse brain atlas was used for reference, and regions of interest (ROI) were manually defined and mapped using ImageJ as described previuously^104,105^.

### Immunostaining

To assess the expression and localization of α-synuclein and Iba1 in the hippocampus, immunofluorescent staining was performed on paraffin-embedded brain tissue sections. Tissue blocks were sectioned at 5-μm and mounted on charged glass slides. After baking for 60 minutes at 60°C, the slides were deparaffinized by immersion in xylene and rehydrated through descending grades of ethanol. Antigen retrieval was performed for 45 min in a decloaking chamber (Biocare) with IHC Antigen Retrieval Solution (Invitrogen; Cat# 00–4955-58). Non-specific binding was blocked with 10% goat serum in phosphate-buffered saline (PBS).

Primary stain was performed with purified mouse anti-α-Synuclein antibody (BD Biosciences, Cat#610787) at a 1:100 dilution, followed by a counterstain with goat anti-mouse IgG secondary antibody-AF488 (Invitrogen, Cat#A11001) at a 2 μg/ml. Similarly, for microglia marker Iba1, sections were incubated with anti-Iba1 antibody (SYSY Antibodies, Cat#234308) at a 1:500 dilution, and counterstained with Goat anti-guinea pig IgG secondary antibody-AF647 (Invitrogen, Cat#A21450) at a concentration of 2 μg/ml. The primary antibodies used in the study was anti- mouse and was used based on previous study using hamster where cross-reactivity across the species was reported^79^.

Whole slide image was captured using Zeiss Axioscan 7 microscope and Zen software (v3.5), images were analyzed using Halo (v3.6.4134.95) image analysis software. Quantification of stained area was performed using Indica Labs Area Quantification FL v2.3.4 module for α-synuclein quantification and Indica Labs – Microglial Activation FL v1.0.6 (activation_process_thickness:1.5) for microglial analysis. Analysis focused specifically on the hippocampal region. The brain sections from 6 hamsters, three control (one old male and two young females) and three infected (one old male and two young females), at 30 days post-infection, were included.

### Western blot analysis of pS129 and total α-synuclein levels

Brain tissue samples were prepared by mechanically homogenizing approximately 50 mg of tissue in 1,000 μL of ice-cold RIPA lysis buffer (Thermo Scientific; Cat#89900) containing protease inhibitors. The mixture was incubated on ice, followed by sonication and was centrifuged (for 20 min at 4°C) to remove debris and obtain a clear supernatant containing soluble proteins. Further the samples were prepared for electrophoresis by dilution in the lysis buffer, addition of 4× NuPAGE LDS sample buffer supplemented with 1.25% β-mercaptoethanol, and boiling for 5 min. Samples were then electrophoresed on NuPAGE 4–12% Bis-Tris gels using NuPAGE MES-SDS running buffer and SeeBlue Plus2 molecular weight markers (all Invitrogen) at 140 V, followed by transfer to nitrocellulose membranes (iBlot 2 NC regular stacks; IB23001) using the iBlot 2 system (Invitrogen). Membranes were fixed for 10 min in 0.4% paraformaldehyde in PBS, blocked for 1 h in 5% milk in TBST, and incubated with primary antibodies overnight at 4°C. After five 5-min washes in TBST, membranes were incubated for 1 h at room temperature with secondary antibodies prepared in blocking buffer, washed again five times for 5 min in TBST, and scanned on an Odyssey CLx (Li-Cor). pS129 to total α-synuclein ratios were calculated and plotted using GraphPad Prism version 10. Primary antibodies used were α-synuclein (BD Biosciences Syn1, 610787; Abcam MJFR1, ab138501), α-synuclein pS129 (Abcam EP1536Y, ab51253; Cell Signaling D1R1R, 23706S), calnexin (Sigma C4731), GAPDH (Abcam 6C5, ab8245), PGK1 (Abcam ab199438), and β3-tubulin (BioLegend; 801202); secondary antibodies were anti-rabbit IRDye 800CW and anti-mouse IRDye 680RD (Li-Cor).

### Microbiome Analysis

Approximately 200 mg of fecal sample was used for DNA extraction using a spin column-based Stool DNA Isolation Kit (Norgen Biotek Corp., Cat#27600), following the manufacturer’s instructions. Extracted DNA was shipped on dry ice to LC Sciences, LLC (Houston, TX, USA) for 16S rRNA gene sequencing. Library preparation involved amplification of the V3–V4 variable regions of the 16S rRNA gene, followed by the addition of sequencing adapters and barcodes after the first PCR cycle. Sequencing was performed on the NovaSeq 6000 platform using a 2 × 250 bp paired-end format (NovaSeq 6000 SP Reagent Kit, 500 cycles). An average of 83,785 paired-end reads was obtained per sample. However, for consistency and quality considerations, raw 16S rRNA gene sequencing reads were processed using the DADA2 pipeline (v1.36.0) and phyloseq (1.52.0) packages in R as previously described with slight modification^106^. Quality filtering and trimming were applied to low-quality reads, and primer contaminants were removed^107^. Error rates were learned from the sample sequence and used to denoise the entire dataset. Amplicon Sequence Variants (ASVs) were then inferred using a denoising algorithm, which corrects sequencing errors and resolves true biological sequences at single-nucleotide resolution. The resulting ASV were merged across all samples to generate a unified feature table. During this process, chimeric sequences were removed to ensure that only high-confidence, non-artifactual ASVs were retained for downstream analyses. Quality-filtered deionized sequence reads were imported into a phyloseq object for downstream ecological analysis.

Taxonomy classification of the ASV was performed using the assign Taxonomy function in DADA22, with SILVA v138.2 training set as the reference^108^. A phylogenetic tree was constructed using Analyses of Phylogenetics and Evolution (ape) package (v5.8.1), and the data were combined into a unified phyloseq object using the phyloseq package (v1.52.0), integrating ASV, abundance, taxonomy, sample metadata, and phylogenetic relationships. Alpha diversity, which measures the richness and evenness of microbial communities within each sample, was calculated from the DADA2 output. The resulting ASV table was converted into a phyloseq object. Diversity metrics, like Shannon, Simpson (richness and evenness), Chao1, and Fisher (richness estimators) were analyzed using the estimate_richness function in the phyloseq package and visualized using ggplot2 (v3.5.2).

Beta diversity was evaluated using the Bray–Curtis dissimilarity index^109^. Bray–Curtis distances were calculated using the phyloseq package in R, and patterns of community dissimilarity were visualized using a heatmap. For community composition analysis, relative abundances were calculated through total-sum scaling and aggregated at multiple taxonomic ranks using the tax_glom function, and taxa with very low abundance (<0.01%) were grouped as “Other” for clarity^110^. Mean relative abundance per group was calculated and visualized using ggplot2 (v3.5.2), enabling comparison of microbial composition. Furthermore, we performed distance-based redundancy analysis (dbRDA) to investigate how gut microbiota community composition differs at both genus and family level, using the vegan (2.7.1) package in R^111^. Using the data from relative abundance analysis, samples were agglomerated to genus and family levels, and constrained ordinations were run for each pairwise comparison. The top 20 taxa contributing to separation along the first canonical axis (CAP1) were identified and plotted using ggplot2 (v3.5.2)^112^.

### Statistical Analysis

Comparison of SARS-CoV-2 viral genomic RNA levels between groups was performed using the non-parametric Mann-Whitney U test. Significant difference between the two groups for radiological analysis (fractional anisotropy and mean diffusivity) was determined using an unpaired t-test. For marble burying behavior measured over time, the Friedman test followed by Dunn’s multiple comparisons was used. Analysis of α-synuclein levels was conducted using one-way Brown-Forsythe and Welch’s ANOVA with Dunnett’s T3 post hoc to correct for heterogeneity in variances and unequal sample sizes. Statistical analysis was performed using Microsoft Excel (v 2108) and/or GraphPad Prism (v10).

### Ethics statement

All infectious work with SARS-CoV-2 was carried out in biosafety level 3 laboratory facilities at Durham Research Center, University of Nebraska Medical Center (UNMC), under the supervision of trained veterinary personnel. All the SARS-CoV-2-infected animals were kept in biosafety level 3 facilities in compliance with the National Institutes of Health’s Guide for the Care and Use of Laboratory Animals and the Guidelines to promote the welfare of animals used for scientific reasons. The study was approved by UNMC Institutional Animal Care and Use Committee (protocol # 21–019-06-FC).

## Limitations

This study has some limitations that worth mentioning. While the GSHs recapitulate many aspects of COVID-19 and Long COVID, they cannot fully capture the complexity of human COVID-19 and disease progression to PASC, including comorbidities, genetic diversity, and environmental exposures. Additionally, herein, only the Omicron variant was examined, and outcomes may vary with other viral strains. Behavioral, imaging, and transcriptomic data were presented for two time points, which may not capture the entire spectrum of the disease pathogenesis post recovery from acute illness. Future studies incorporating longer follow-up periods, inclusion of multiple strains of SARS-CoV-2 circulating in the community, and cross-species validation of primary study endpoints with humans, will help to identify therapeutic targets for prevention and or treatment of Long-COVID.

## Supplementary Material

Supplementary Files

This is a list of supplementary files associated with this preprint. Click to download.

• KumarNetalnrreportingsummary.pdf

• Supplementarymaterials.pdf

• RNA.docx

## Data Availability

Supplementary Tables 1 and 2 depicts the details of samples sequenced from each group. The transcriptomic data for brain and lung is uploaded to SRA via the Bio project ID PRJNA1196444. The Heart transcriptomic data was uploaded to SRA via the Bio project ID PRJNA1335562. 16S rRNA sequencing files have been deposited in the NCBI Sequence Read Archive with the Bio project ID PRJNA1337707.
